# Metastatic Behavior in Melanoma: Timing, Pattern, Survival, and Influencing Factors

**DOI:** 10.1155/2012/647684

**Published:** 2012-06-27

**Authors:** Faruk Tas

**Affiliations:** Institute of Oncology, Istanbul University, 34390 Istanbul, Turkey

## Abstract

Metastatic melanoma (MM) is a fatal disease with a rapid systemic dissemination. This study was conducted to investigate the metastatic behavior, timing, patterns, survival, and influencing factors in MM. 214 patients with MM were evaluated retrospectively. Distant metastases (82%) were the most frequent for patients initially metastatic. The median and 1-year survival rates of initially MM patients were 10 months and 41%, respectively. The median time to metastasis for patients with localized disease was 28 months. The timing of appearance of metastases varied minimally; however, times to metastases for distant organs varied greatly. For the first metastatic pathway, more than half of the primary metastases were M1A (57%). These findings were in contrast to the results compared with those with metastatic in diagnosis (*P* < 0.001). The median and 1-year survival rates of all patients were 12 months and 49%, respectively. Outcome was higher in M1A than visceral metastases (*P* < 0.001). In conclusion, the fact that over half of all recurrences/metastases occurred within 3 years urges us to concentrate follow-up in the early time periods following diagnosis. Because the clinical behavior of MM is variable, the factors for survival consisting of site and number of metastases should be emphasized.

## 1. Introduction

Over several decades, the incidence of melanoma has steadily risen as incidence rates have increased by averages ranging from 3 to 8% per year and continue to rise nowadays. Melanoma represented estimated 5% and 4% of incidents of cancer in males and females in 2009 and it was the fifth and sixth most common type of cancer in males and females, respectively [1]. Now, presents a lifetime risk of one in 39 for men and one in 58 for women in the USA.

Melanoma is an aggressive and highly metastatic disease. Metastatic melanoma is a fatal disease with a rapid systemic dissemination. The 5-year survival rate is less than 15% in patients with metastatic disease. While the minority of patients, which constitute 4% of newly diagnosed melanoma patients [1], present with distant metastasis at initial diagnosis, the majority who present with early stage initially eventually develop metastatic disease as a consequence of disease progression. Approximately one-third of all melanoma patients will experience disease recurrence [2]. Although almost all organs can be involved, the most frequent target sites are the liver, bone, and brain. Despite recent advances in the understanding of oncogenic mechanisms and therapeutic interventions, the median survival in patients with metastatic disease does not go beyond 12 months. 

In spite of the fact that factors that predict recurrence have been well described, few studies have investigated the natural history of melanoma, including factors that determine and influence type, pattern, and timing of recurrence/metastases of melanoma [2–6]. In this retrospective study, we analyzed the metastatic timing, patterns, and factors influencing metastasis and survival in patients with metastatic melanoma.

## 2. Material and Methods

Two hundred and fourteen patients with histologically confirmed melanoma, treated in our clinic with metastatic disease from 1997 to 2009, were evaluated retrospectively; 66 patients presented initially with metastatic disease, while 148 patients with early stage developed metastasis during treatment or follow-up.

All patients were evaluated and staged at the first visit by history, physical examination, CBC, serum biochemistry analysis, chest X-ray and CT imaging, cranial CT or MRI, whole-body scan and abdominal CT or USG (ultrasonography). The standard follow-up protocol was applied for all of the patients.

Time to metastasis was defined as the time period from the date of histological diagnosis to the time of appearance of metastasis. Overall survival was determined as the time elapsed between the time of histological diagnosis and the date of death or the last follow-up visit. The period from the date of relapse to death or the last follow-up day was referred to as the postrecurrence survival.

*χ* ^2^ tests were performed to test differences of frequencies. Overall and postrecurrence survival values were analyzed by the Kaplan-Meier method. Univariate analyses were performed by the log-rank tests. Statistical differences were accepted as significant at *P* < 0.05.

## 3. Results

The median age of 214 patients was 50 years (range: 22 to 86), and there was a predominance of males, 62% of subjects were men. Patient characteristics are summarized in Table 1. Sixty-six patients presented initially with metastatic disease, while 148 developed metastatic disease during follow-up for early stages. The distribution of metastatic involvement is shown in Table 2.

### 3.1. Metastases at Presentation

Totally 66 patients (31% of all patients) were diagnosed with metastatic disease at presentation. 

The characteristics of the patients are shown in Table 1. The median age was 50 years, two-thirds of the patients were men, and their primary lesions were more axial sited (56%). The distributions of clinicopathologic forms (nodular versus nonnodular) and Breslow thickness (less versus more than 4 mm) were identical.

Slightly more than half (56%) of the patients presented with single metastases (Table 2). The distant metastases, 82% totally, combined with 67% for M1C and 15% for M1B, were the most frequent for initially metastatic patients, and distant skin, subcutaneous, or nodal metastases (M1A) were the least common (18%). For the involvement of distant organ other than the lung (M1C), liver was the most common site (48%) followed by the brain (29%) and bone (23%).

The distribution of metastatic behaviors, such as the number and site of metastases, was not different in relation to the parameters of the tumors and the patients. 

**Figure 1:**
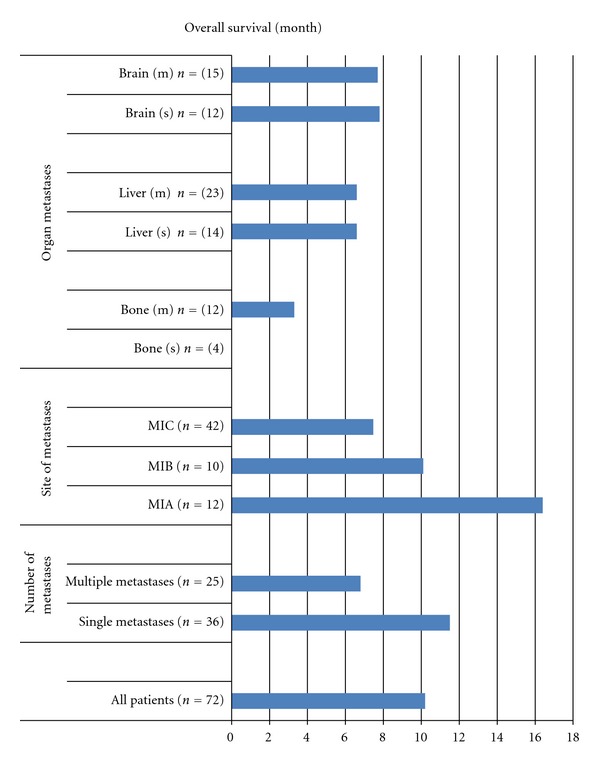
Overall survival rates of metastatic patients diagnosed at presentation. S: only single-organ metastasis; M: mixed with other distal organ metastases.

The median and 1-year survival rates of initially metastatic patients were determined as 10 months and 41%, respectively (Figure 1). Survival in patients with single metastasis was higher than that in those with multiple metastases (median 12 versus 7 months, resp., *P* = 0.07). Likewise, as statistically insignificant, the patients with M1A had the higher and those with M1C had the lower survival rates (*P* = 0.2). However, there was no difference in distribution of visceral organ involvement with respect to organ site metastases (*P* = 0.8).

### 3.2. Metastases during Follow-Up

One hundred and forty-eight patients with cutaneous melanoma diagnosed at the stage of the primary tumor without detectable metastases subsequently developed metastases. The characteristics of the patients are shown in Table 1. Regarding patient and disease parameters, no difference was found between the patients who presented with metastases at diagnosis and these patients.

**Figure 2:**
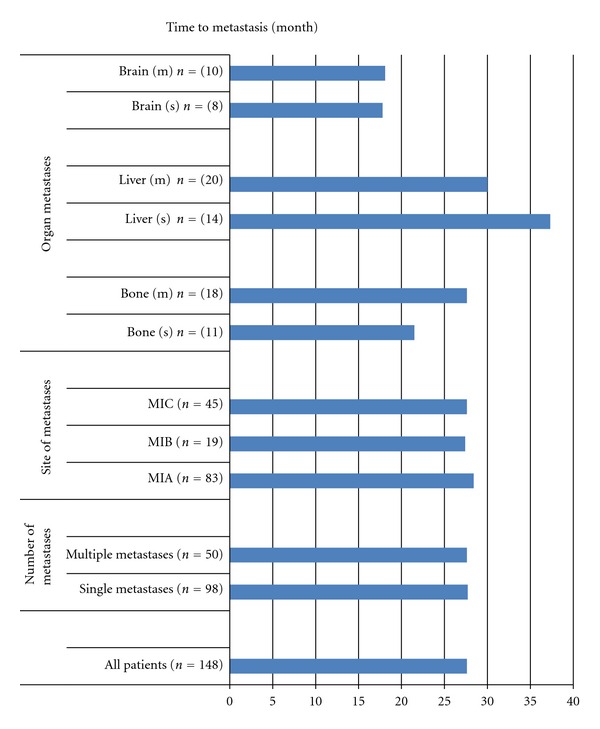
Time periods to metastases in metastatic patients with diagnosed during follow-up.

The median time to metastasis for the 148 patients with localized disease who developed metastases during follow-up was 28 months (Figure 2). The timing of appearance of metastases according to metastatic M1 stage varied minimally. Thus, the median times to metastases were 28, 27, and 28 months for patients with M1A, M1B, and M1C, respectively. Likewise, there is no difference for occurrence time of metastasis between single and multiple metastases. However, times to metastases for distal organs other than the lung varied greatly: the longer times for liver metastases, the shorter times for brain metastases, and the equal values for bone metastases.

Two-thirds of the patients had single metastatic disease (Table 2). The first metastatic pathway in relation to the primary tumor site showed that more than half of the primary metastases were distant skin, subcutaneous, or nodal metastases (M1A) (57%) followed by distant metastases other than the lung (M1C) (30%) and lung metastases (M1B) (13%). These findings were contrast to the results compared with those with metastatic in diagnosis (*P* < 0.001). The percentages of the liver (42%) and bone (37%) involvement were identical and more than cerebral metastasis (21%). When compared with the group containing the metastatic patients diagnosed at presentation, no difference was found.

**Figure 3:**
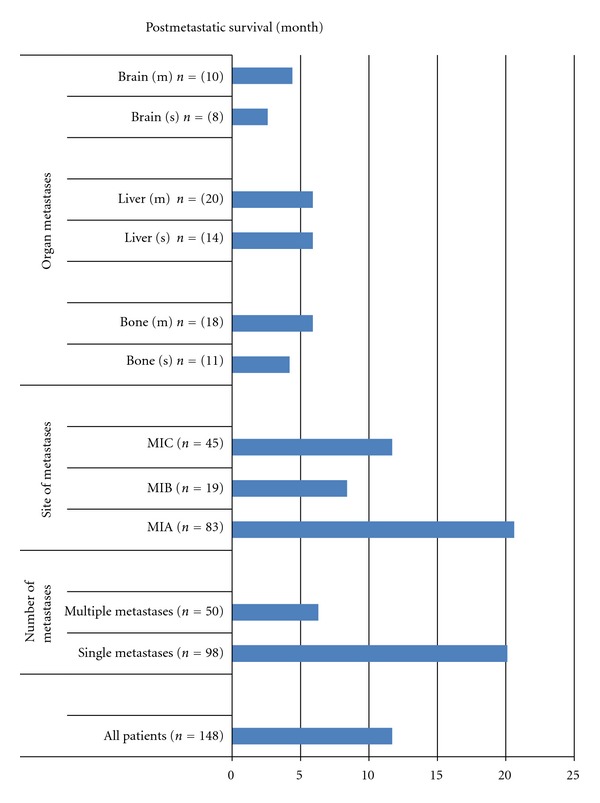
Postmetastatic survival values in metastatic patients diagnosed during follow-up.

The median survival and 1-year survival rates of all patients were 12 months and 49%, respectively (Figure 3). Single-organ metastasis showed significant survival advantages compared with multiple metastases (median 20 versus 6 months, resp., *P* < 0.001). Similarly, overall survival was found to be significantly higher in M1A than in M1B and M1C (*P* < 0.001). However, in contrary to this, there was no difference among distant organs with respect to site of organ involvement.

### 3.3. Secondary Metastases

In our study, 72 out of total 214 (34%) patients with metastatic melanoma went on to develop metastases twice during follow-up. With respect to gender and age of the patients, there was no difference among patients. Interestingly, patients with axial localization of primary melanoma (92%) presented mostly than those with extremity sites (*P* < 0.001).

**Figure 4:**
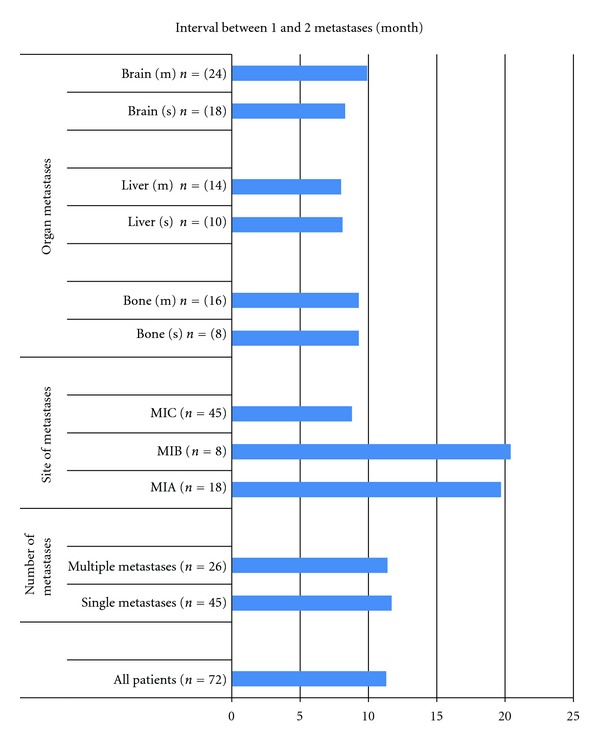
Intervals from the date of first metastases to the date of second metastases in metastatic patients who presented twice with metastatic disease.

The median time to development of second metastases from the time of first metastases was 11 months (Figure 4). The timing of appearance of metastases according to metastatic M1 stage varied greatly. Thus, the median times to development were 20 and 21 months for M1A and M1B, respectively, and M1C had highly lower times (9 months). However, in contrast, there were no differences for occurrence times among the M1C organs. 

When we look at the occurrence from first metastases to second metastases, more transformations occurred among M1A to M1C (32%), M1A to M1A (21%), M1B to M1C, (17%), and M1C to M1C (15%) of patients (Table 3). The development of M1C prominently occurred in consequence of 51% for M1A, 86% for M1B, and 85% for M1C.

**Figure 5:**
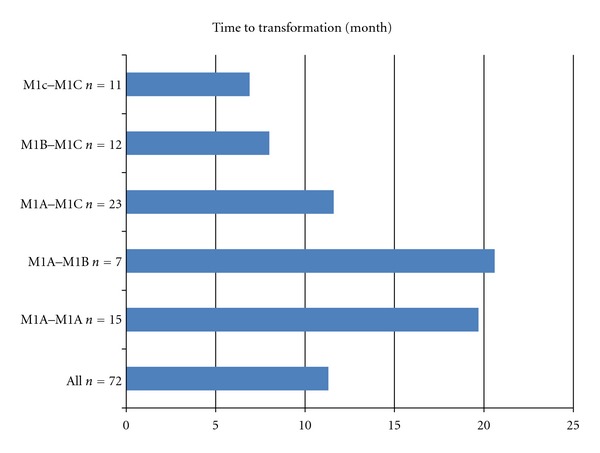
Intervals between initial metastases and secondary metastases.

The timing of appearances of metastases according to metastatic transformation varied The median times to secondary metastases development were 20 and 21 months for M1A to M1A and M1A to M1B, respectively, and 7 months for M1C to M1C (Figure 5). 

**Figure 6:**
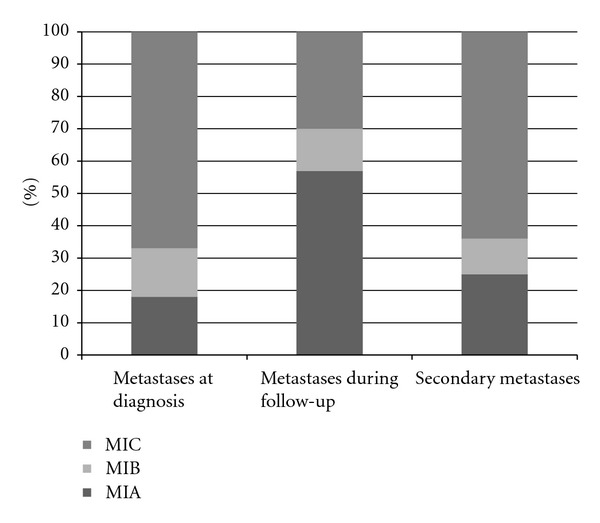
The distributions of M stages depend on time of metastasis presentation (%).

Similar to former groups, the metastatic distribution was single metastatic area in nearly two-thirds of patients (Table 2). Identical to the patients with metastases at presentation, 75% of the patients who developed clinical metastases secondarily presented with distant metastases, combined with 64% for M1C plus 11% for M1B and distant skin, subcutaneous, or nodal metastases (M1A) developed in only one-fourth of patients (Table 2). In other words, these findings were in contrast with the data in metastases during follow-up (*P* < 0.001) and similar to the data regarding metastases at presentation (Figure 6).

**Figure 7:**
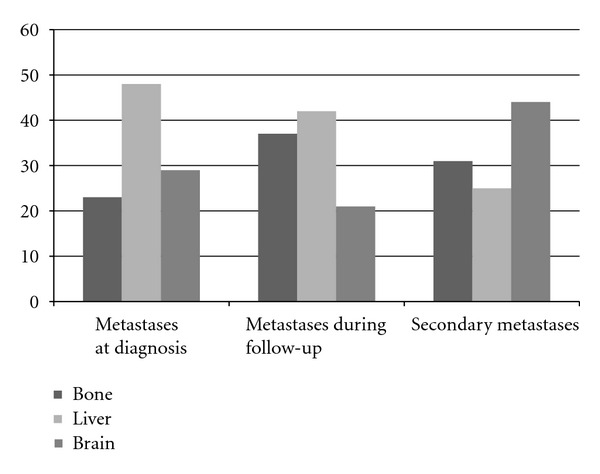
The distributions of distant organs depend on time of metastasis presentation (%).

In 44% of the patients the second metastases during follow-up developed in the brain. In the remaining half of the patients, bone (31%) and liver (25%) involvement carried out (Table 2). These values are statistically different from both metastasis at presentation (*P* = 0.05) and metastasis during follow-up (*P* = 0.04) (Figure 7). While bone metastases were distributed equally with respect to gender, liver (64% versus 36%, *P* = 0.02) and brain (79% versus 21%, *P* = 0.04) metastases were found more in females and males, respectively. No other correlation was found between the factors of patient/disease and the number/site of the metastases.

## 4. Discussion

Patients with metastatic melanoma generally have a poor prognosis; survival is limited and typically measured in months rather than years. In general, the duration of survival is less than a year, a median of nearly 6 to 8 months. The 1-year survival rate is 45%, and less than 10% will live for 5 years or more. Multivariate analyses of prognostic factors have identified several independent factors that predict survival in this poor prognosis group, including the site of the first metastases, number of metastatic sites, and duration of remission [2–4].

The site of distant metastasis is an important independent predictor of survival in patients with metastatic disease [3, 4]. In the 2002 AJCC melanoma database analysis, the greatest difference in survival was found showing that patients with locoregional, distant nodal, and soft tissue metastasis have a better survival rate than the patients with visceral metastasis [3, 7]. Additionally, patients in whom the lung was the only site of visceral metastasis had a better 1-year survival duration time compared with those with metastasis in other visceral sites. In the recent analysis of the AJCC melanoma database, separation of patients into three groups based on sites of disease produced the greatest splay in median survival [3, 8]. Patients with melanoma metastasis to visceral sites other than the lung (M1C) had a median survival of 7 months, those with lung metastases had a median survival of 12 months, and those with metastasis to nonvisceral sites (i.e., skin, subcutaneous tissue, and distant lymph nodes) had a median survival of 18 months. In general, patients who have visceral metastases to sites other than the lung, such as the liver, brain, or bone, do poorly with a median survival ranging from 3 to 6 months.

Patients with one distant metastatic site have a significantly improved outcome compared with those with two or more distant sites [3]. The number of metastatic sites was the most significant prognostic factor in patients with distant metastasis [9]. In this study, patients with one, two, three, or more sites of distant metastasis had a median survival duration of 7, 4, and 2 months, respectively. The 1-year survival rate was 36% for patients with one metastatic site, 13% for patients with two sites, and less than 1% for patients with three or more sites. In another study, the number of metastases, one versus more than one, was the strongest independent predictor of survival; the median survival times for groups were 23 months and 8 months, respectively [10]. In the 2008 stage IV collaborative melanoma database preliminary analysis, the number of metastatic sites was also associated with survival by both univariate and multivariate analyses [8]. In contrast to these studies, however, the number of metastatic sites was not a significant independent prognostic factor in the multivariate analysis of the studies [11]. This may suggest that certain sites of metastases have a dominant negative effect on survival. Patients whose initial site of metastases was the liver or brain had a median survival of only 4 months compared with patients whose initial sites were the skin and/or lymph nodes, who had a median survival of 15 months [4, 11].

Few patients with newly diagnosed melanoma have clinically evident distant metastases at the initial diagnosis [1, 4]. The majority of the metastatic patients who present with early stage initially eventually develop metastatic disease as a consequence of disease progression. Nearly one-third of all melanoma patients will experience disease recurrence [2]. For most patients without distant metastases, the time to recurrence/metastasis varies inversely with tumor stage at presentation. For patients with thicker tumors the risk of recurrence is the greatest in the first year after treatment and declines steadily over time [2, 4]. Most recurrences (55% to 79%) become evident by 2 years, whereas 65% to 85% are apparent by 3 years after the initial diagnosis of the primary tumor. In addition, the disease-free interval is considerably shorter in patients with ulcerated tumors [2, 4]. In general, patients with nodal metastases, stage III, have recurrences earlier than patients whose lymph nodes are negative. In addition, age of diagnosis can also influence the timing of distant metastases, that is, patients older than 50 years of age have been shown to relapse sooner than younger patients. The disease-free interval before the onset of distant metastasis was a significant prognostic factor in a multivariate analysis of studies [9, 11]. The stage of disease preceding distant metastasis was also identified as an important prognostic factor [11]. For patients who progressed directly from stage I or II disease, a disease-free interval of 34 months or longer was associated with prolonged survival, whereas for patients with a history of stage III melanoma, a disease-free interval of 18 months or longer was associated with prolonged survival.

Melanoma is well known for its ability to metastasize to virtually any organ or tissue, including some sites rarely seen with other solid tumors [4]. Nevertheless, some sites are more likely to harbor initial distant metastases. The initial sites of distant metastases are most commonly the skin, subcutaneous tissue, and lymph nodes, which occurred in 42% to 59% of patients in various studies. Visceral metastases were the initial sites of relapse in approximately 25% of all metastatic melanoma patients. The most common sites of visceral metastases were the lung (18–36%), brain (12–20%), liver (14–20%), and bone (11–17%).

In conclusion, lifetime follow-up of melanoma patients, particularly during the first three years, is necessary because the expected cure is rarely achieved after surgical excision and also given adjuvant treatment. The fact that over half of all recurrences/metastases occurred within 3 years urges us to concentrate follow-up in the early time periods following diagnosis. Generally, the prognosis of patients with metastatic melanoma is poor; however, because the clinical behavior of metastatic melanoma is variable, significant factors for survival consisting of site of metastasis and number of metastatic sites should be emphasized.

## Figures and Tables

**Table 1:** Patient's characteristics.

Parameter	Metastasis at presentation (%)	Metastasis during follow-up (%)
	31	69
Gender		
Male	65	61
Female	35	39
Age		
Median (range)	50 (22–86)	49 (24–83)
Localization		
Axial	56	58
Extremity	44	42
Histology		
Nodular	50	51
Nonnodular	50	49
Breslow thickness		
<4mm	50	51
>4mm	50	49
Clark invasion		
I–III	5	18
IV-V	95	82
Ulceration		
No	36	17
Yes	64	83
Mitotic rate (/mm^2^)		
Median (range)	3 (1–46)	4 (1–52)
Lymphocyte infiltration		
No	47	40
Yes	53	60
Regression		
No	83	85
Yes	17	15
Vascular invasion		
No	71	82
Yes	29	18
Neurotropism		
No	89	93
Yes	11	7

**Table 2:** Metastatic patterns of cases.

	Metastasis at presentation 66 (31%)	Metastasis during follow-up 148 (69%)	Secondary metastasis 72 (34%)
Number of involvements			
Single	37 (56%)	98 (66%)	45 (63%)
Multiple	29 (44%)	50 (34%)	27 (37%)

Stage IV			
M1A	12 (18%)	84 (57%)	18 (25%)
M1B	10 (15%)	19 (13%)	8 (11%)
M1C	44 (67%)	45 (30%)	46 (64%)

Stage M1C			
Bone	12 (23%)	18 (37%)	17 (31%)
Liver	25 (48%)	20 (42%)	14 (25%)
Brain	15 (29%)	10 (21%)	24 (44%)

**Table 3:** Transformations of metastases from first to second.

From	To		
	M1A	M1B	M1C
M1A (*n* = 45)	15 (33%)	7 (16%)	23 (51%)
M1B (*n* = 14)	1 (7%)	1 (7%)	12 (86%)
M1C (*n* = 13)	2 (15%)	0 (0%)	11 (85%)
